# Interval between Removal of a 4.7 mg Deslorelin Implant after a 3-, 6-, and 9-Month Treatment and Restoration of Testicular Function in Tomcats

**DOI:** 10.3390/ani10091559

**Published:** 2020-09-02

**Authors:** Lluis Ferré-Dolcet, Lisa Carniello, Silvia Ferro, Andrea Cattai, Stefano Romagnoli, Antonio Mollo

**Affiliations:** 1 Department of Animal Medicine, Production and Health, University of Padova, 35020 Padova, Italy; stefano.romagnoli@unipd.it (S.R.); antonio.mollo@unipd.it (A.M.); 2 Private Practitioner, 36403 Camisano Vicentino, Italy; carniello.lisa@gmail.com; 3 Department of Comparative Biomedicine and Food Science, University of Padova, 35020 Padova, Italy; silvia.ferro@unipd.it; 4 Private Practitioner, 35020 Padova, Italy; andrea.cattai.vet@gmail.com

**Keywords:** deslorelin, testosterone, reproductive function restoration, breeding

## Abstract

**Simple Summary:**

Gonadotropin-releasing hormone (GnRH) agonists bind to GnRH receptors in the anterior pituitary causing an initial release of luteinizing hormone (LH) and follicle-stimulating hormone (FSH) followed by a desensibilization of the GnRH receptors. On the first days of treatment, this results in an increase in testosterone secretion in males, while in females, it may induce an estrous cycle. This is commonly referred to “flare-up effect” and its duration depends on the active substance and the dose. After this initial stimulation, pituitary GnRH receptors are downregulated with a consequent decrease in ovarian and testicular activity. In tomcats, researchers found that testosterone levels rapidly decline 20 days after the insertion of a 4.7 mg deslorelin implant, remaining at basal levels (below 0.1 ng/mL) for a prolonged period of time. However, there is a high individual variability in the response to treatment, and the duration of efficacy varies between 6 and 24 months. As a consequence of basal testosterone levels, testicular volume decreases, and sexual behavior and penile spines disappear. All of these effects are completely reversible once the implant is not active any longer or following implant removal.

**Abstract:**

Deslorelin implants have been used to produce a reversible sterilization in several species. In cats, the prolonged duration (12–15 months in tomcats and 18–22 months in queen) is often too much for cat breeders who request early implant removal. The interval between implant removal and resumption of reproductive function in cats has never been investigated. Eighteen tomcats received a 4.7 mg deslorelin implant placed in the periumbilical area and surgically removed during all seasons of the year after 3, 6, or 9 months (n = 6, 6, and 6 cats, respectively). Following implant removal, all cats received a clinical exam every two weeks, including testicular ultrasonographic measurement, observation of penile spikes, and blood collection for serum testosterone assay. Restoration of serum testosterone secretion occurred after 23 ± 6, 23 ± 6, and 22 ± 7 days in the 3-, 6-, and 9-month groups, respectively. Restoration of testicular function was confirmed by histology in 13/15 cats undergoing orchiectomy at the end of the study while the owners of the remaining two cats opted to maintain their animals intact. Removal of a 4.7 mg deslorelin implant after 3, 6, or 9 months is followed by resumption of serum testosterone secretion after about 3 weeks independent of age or season.

## 1. Introduction

Deslorelin is a gonadotropin-releasing hormone (GnRH) superagonist 100 times more potent than endogenous GnRH [1]. Deslorelin is marketed as subcutaneous implants for use in male dogs to cause a reversible chemical sterilization through a desensibilization of hypothalamic GnRH receptors, which suppress pituitary gonadotropin secretion [2]. In male dogs, deslorelin causes a lowering of serum testosterone (T) concentration to undetectable levels, reduction of testicular volume and lack of sperm production from 6–8 to 24 weeks post-treatment [3,4,5,6]. A similar reversible long-term contraception was observed following the use of deslorelin implants in many species, such as ferrets, cats, wild carnivores, birds, and marsupials [7,8,9,10,11]. In cats, administration of a 4.7 mg deslorelin implant caused an initial increase in T concentration and peaking 2 days later [12]. Thereafter, serum testosterone of treated cats undergoes a rapid decline to <1.0 ng/mL persisting around basal levels for 61.7 to 100.7 weeks [12,13]. The interval between implant administration and the decrease of reproductive hormones has been defined as “time to downregulation” [14]. Such downregulation is reported to occur within 10 or 16 weeks in 90% [14,15] and 100% [16] of tomcats, respectively, and is accompanied by behavioral (decreased roaming and urine marking), physical (decrease in testicular size, disappearance of penile spike) and histological (suppression of spermatogenesis) changes [12,16,17,18,19]. Because of these relevant and reversible changes in fertility, the off-label use of deslorelin in cats has acquired importance among cat breeders, as temporarily reproductive suppression of a tomcat in a breeding establishment may be useful when there is more than one male (to prevent fighting or alternate the use of males), or to prevent excessive weight loss in show tomcats during the breeding season. However, the long duration of action (12–15 months) of deslorelin implants in tomcats makes the use of this compound often inconvenient, as most breeders would like their tomcat’s fertility to be restored sooner.

Early surgical removal of deslorelin implants may offer a solution for cat breeders. When a deslorelin implant was removed from 8 tomcats treated 4 months previously, a gradual return to normal testosterone secretion and normal reproductive behavior was observed starting from the fourth week after surgical removal [16]. However, there is a lack of information on interval from surgical removal to resumption of gonadal function in cats treated for variable durations of time. Breeders often ask for a fertility check in their tomcats when restoration of full fertility following implant removal is delayed, which may cause cats to undergo unnecessary hormonal assays and semen collections. Therefore, the aim of this study was to evaluate the interval between removal of a 4.7 mg deslorelin implant left in situ for 3, 6, or 9 months, and resumption of serum testosterone secretion in adult tomcats.

## 2. Materials and Methods

### 2.1. Selection of Animals

The current study was approved by the University of Padova Ethics Committee (Project n. 323548). Privately owned tomcats were recruited with owner’s consent to be treated with a subcutaneous 4.7 mg deslorelin implant (Suprelorin™ Virbac, Carros, France) among the patients of the Veterinary Teaching Hospital of the University of Padua, Italy. Reproductive, social and behavior data from each animal were collected by means of a questionnaire. Requirements for inclusion in the study were to be (a) a sexually mature tomcat showing secondary sexual characteristics and behavior (urine marking, tomcat urine odor, penile spikes) presented for temporary suppression of fertility, and (b) in good general health conditions (including testing negative to feline immunodeficiency and feline leukemia virus).

Upon signing the informed consent, cat owners were given a choice of including their tomcat in the 3-, 6-, or 9-month duration of treatment, following which the implant would be surgically removed. Testicular histology was assessed in case orchiectomy was requested by the owner following the end of the study. The present study was designed as a non-randomized, non-blinded study.

### 2.2. Serum T Determination

Blood samples were collected from the jugular vein. Serum T was assayed prior to deslorelin administration by a stimulation test with gonadorelin (Fertagyl™; Intervet, Milan, Italy), 50 μg (total volume 0.5 mL) administered intramuscularly in the posterior limb 60 min prior to sampling [20]. Assay of serum T concentration was also performed after half time of treatment, at day of implant removal and every two weeks after implant removal until gonadal steroid hormone secretion was restored. Serum T concentration was determined by chemiluminescence (Coefficient of variation: 16%) (Immulite 1000; Siemens, Milan, Italia) [17].

### 2.3. Semen Collection and Evaluation

Prior to deslorelin treatment and on the day of orchiectomy at the end of the study, semen collection was performed under sedation using a dose of 50 μg/kg of dexmedetomidine (Dexdomitor™; Zoetis, Rome, Italy) as previously reported [21]. As soon as tomcats were sedated, a 3-Fr cat urinary catheter was inserted 9 cm deep into the urethra, kept 40 seconds in that position, and then removed. At the end of the procedure, atipamezole (Antisedan™; Zoetis, Rome, Italy) was administered intramuscularly to reverse the α_2_-adrenergic receptor agonist effects. Semen was deposited in a pre-warmed (37 °C) 1.0 mL Eppendorf™ (Milan, Italy) vial, and immediately evaluated using light microscopy (40x) to ensure the presence of motile spermatozoa.

### 2.4. Deslorelin Treatment

Deslorelin implants were placed subcutaneously in the periumbilical area, 1.5 cm cranial to the umbilical scar following clipping and scrubbing. Placement of deslorelin implants in the periumbilical area has been reported and is thought to allow for easier localization and faster removal of implants [17,22].

### 2.5. Follow-Up after Implantation

Every tomcat was checked once during treatment. Evaluation was performed after approximately 50% of the treatment period had elapsed (as part of our routine sequence of health checks in cats implanted with deslorelin) and included a clinical examination, abdominal, and testicular ultrasound including measurement of testicles, and collection of a blood sample 60 min after the gonadorelin stimulation test for serum T assay.

### 2.6. Deslorelin Implant Removal

Implant removal was performed surgically 3, 6-, or 9-months following implantation. Removals occurred throughout the year and were equally divided between seasons of increasing and decreasing photoperiods. Tomcats were anaesthetized with 0.008 mg/kg of dexmedetomidine, 2 mg/kg of ketamine (Imalgene 1000™; Merial, Padova, Italy) and 0.3 mg/kg of butorphanol (Dolorex™; MSD, Rome, Italy), administered by intramuscular injection and propofol (Proposure™; Merial, Padova, Italy) administered intravenously to effect when required. Once the implant was located by palpation of the periumbilical skin, a 2 cm incision was performed just above the implant following disinfection and scrubbing of the surgical area. Subsequently, the implant was gently pulled out taking care not to rupture it. Following removal, the incision was closed with an intradermal suture using absorbable material (3/0 Monosyn™; Braun, Milan, Italy). At time of implant removal, a GnRH stimulation test (administering 50 μg gonadorelin prior to surgery and collecting a blood sample 60 min later) and an ultrasound evaluation with measurement of both testicles were performed.

### 2.7. Follow-Up after Removal

Follow-up checks were performed through a clinical exam every 14 days following implant removal until restoration of testicular hormone secretion (T > 0.1 ng/mL). Clinical checks included a visual inspection of the surgical wound, of the penis for presence of spikes and of testicles for size and consistency, a testicular ultrasound and a GnRH stimulation test. Owners were reminded about T-related behavioral signs and advised to ask for an appointment if the cat started to show any of those signs in order to time restoration of T secretion. Once restoration of gonadal steroid hormones was confirmed (T > 0.1 ng/mL), orchiectomy was performed.

### 2.8. Measurement and Histologic Evaluation of Testicles

Testicular ultrasonography was performed with a 3.5–5 MHz microconvex probe (Philips Affinity 50 G; Philips, Milan, Italy). Testicular volume was calculated using Hansen’s formula: Volume (V) = length (L) × width (W) × height (H) × 0.71 [23,24]. Orchiectomy was performed within 2 weeks following detection of high levels of serum T (>0.1 ng/mL). Following removal, testicles were fixed in a 4% paraformaldehyde solution. From each formalin-fixed, paraffin-embedded specimen 4-μm-thick sections were obtained with a rotary microtome (RM2145, Leica, S.P.A., Milan, Italy) stained with hematoxylin and eosin using an automated stainer (Leica, autostainer XL), and evaluated under light microscopy (Olympus BX-40; Olympus, Segrate, Italy).

### 2.9. Statistical Analysis

Data were evaluated using a statistical package (SAS 9.4, SAS Institute Inc., Cary, NC, USA). Prior to statistical evaluation, data were tested for normality of distribution (Shapiro-Wilk test). Normally distributed data were analyzed using a linear model which included a fixed effect of treatment (3, 6, and 9 months), season of implant removal (1 = increasing photoperiod; 0 = decreasing photoperiod) and interval of implant removal—detection of serum testosterone as a covariate. In case of non-normally distributed data, these were analyzed using a non-parametric Kruskal–Wallis test. As implant rupture and subcutaneous fat embedding were unexpected outcomes, we decided to investigate potential correlations between implant rupture and subcutaneous fat enclosure using Pearson correlation; moreover, implant rupture and abdominal fat enclosure were confronted with treatment length using an χ^2^ test, setting the level of significance at *p* ≤ 0.05.

## 3. Results

A total of 18 cats (2 Persian, 1 Ragdoll, 1 Maine Coon, 1 Sacred cat of Burma, 2 Sphinx, 2 British shorthair, 9 European) were enrolled for the study, with 6, 6, and 6 cats treated for 3, 6, and 9 months, respectively. Unfortunately, one cat of the 9-month group died suddenly 7 months after being implanted and the owner refused to perform an autopsy. Two cats (one each from the 6- and 9-month group, respectively) escaped from the owner’s facility after the first check and did not come back. Therefore, we have clinical and endocrine data for 15 cats derived from 6, 5, and 4 cats treated for 3, 6, and 9 months, respectively. Two owners did not want their breeding tomcats (belonging to the 9-month duration of treatment) to be neutered and treated them again at the end of the study with another 4.7 mg deslorelin implant. Therefore, we have testicular histology only on 13 cats derived from 6, 5, and 2 cats treated for 3, 6, and 9 months, respectively. Data on pre-treatment testicular volume and its post-treatment reduction were normally distributed, while a non-normal distribution was observed for data relative to the interval from implant removal to resumption of T secretion as well as the post-treatment increase in testicular volume.

### 3.1. Implant Removal

**Figure 1:**
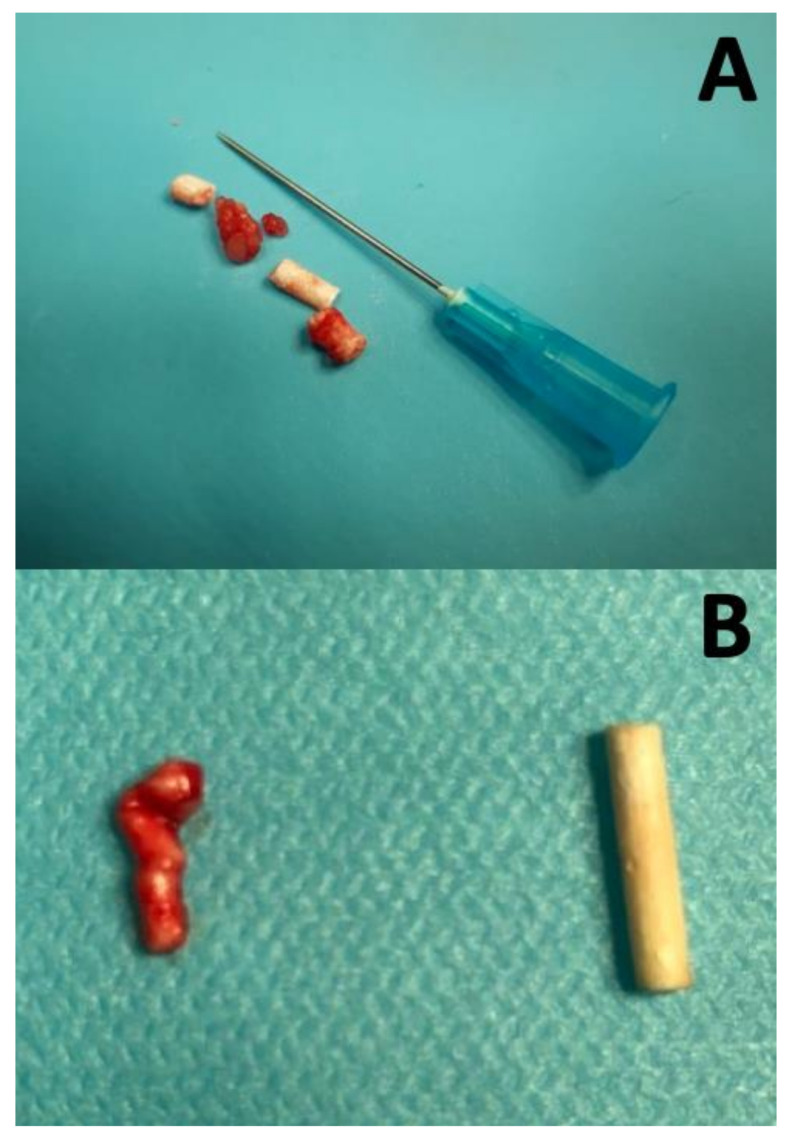
The 4.7 mg deslorelin implants removed surgically from adult tomcats implanted for 3, 6, or 9 months. (**A**) an implant ruptured in four pieces of a cat treated for 6 months; (**B**) comparison between an implant (of a cat treated for 9 months), ruptured and embedded in abdominal fat (left) with an implant of a cat treated for 3 months after surgical removal (right).

All surgical implant removal procedures were uneventful. All implants were found where originally placed, in the subcutaneous tissue approximately 1.5 cm cranial from the umbilical scar with no migration detected. One-third (5/15) of implants were found as a single piece and were easily removed without complications; removal time in this case was 5–8 min. The remaining two thirds (10/15) of implants were found fragmented in two or more pieces (Figure 1A); removal time for these implants varied from 10 to 20 min depending on the number of pieces. In one cat, due to friability of the implant at time of removal, the implant broke in a few pieces when debriding the tissues and the initial pieces could not be retrieved. Moreover, 8/15 implants were found enclosed in subcutaneous abdominal fat, which made their removal slightly longer and more complicated (Figure 1B). All of the implants removed at 9 months were broken in at least 4 pieces. However, the results of both statistical tests were not significant. Data on implant removal are shown in Table 1.

### 3.2. Restoration of Serum Testosterone Secretion

**Figure 2:**
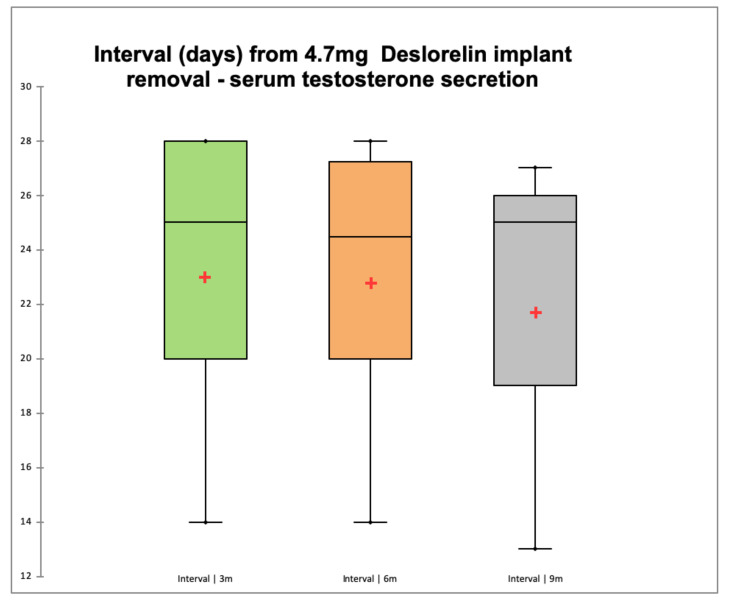
Interval between removal of a 4.7 mg deslorelin implant and restoration of testosterone secretion (≥1.0 ng/mL) in adult tomcats treated for 3 (green), 6 (orange), and 9 months (grey) of 5 and 4 cats treated, respectively. The interval for the three groups of cats was 23 ± 6, 23 ± 6, and 22 ± 7 days, respectively, and did not differ among groups (*p* > 0.05). The red cross indicates the mean value while the black bar above the cross indicates the median value evaluated.

Data are shown as mean ± standard deviation. Interval from implant removal to restoration of serum T secretion did not differ for the 3 treatment groups and was 23 ± 6 (14–28 days), 23 ± 6 (14–28 days), and 22 ± 7 days (13–27 days) for cats treated for 3, 6, and 9 months, respectively (Figure 2). One cat belonging to the 3-month treatment returned to normal serum testosterone secretion at 231 days after removal of the deslorelin implant (which was found ruptured in several pieces) and was excluded from statistical analysis, as it was considered an outlier, being his interval greater than three standard deviations.

### 3.3. Testicular Volume and Consistency

Data on testicular volume are shown on Table 2. Total reduction in testicular volume at time of removal did not differ among treatment groups at 46.61 ± 27.92% (*p* > 0.05). The increase in testicular volume from the day of implant insertion until the day when serum T secretion was restored did not differ among groups at 0.23 ± 0.73% (*p* > 0.05). Testicular consistency at palpation did not vary during treatment.

### 3.4. Body Weight

Data on animal weights are shown on Table 2. Body weight during treatment increased 0.11 ± 0.04 kg (0.95%), 1.93 ± 0.27 kg (33.5%), and 1.66 ± 0.77 kg (47.8%) for cats treated for 3, 6, and 9 months, respectively. Body weight increased significantly more for the 6- and 9-month treatment groups when compared to the 3-month treatment group (*p* < 0.05).

### 3.5. Histological Evaluation

**Figure 3:**
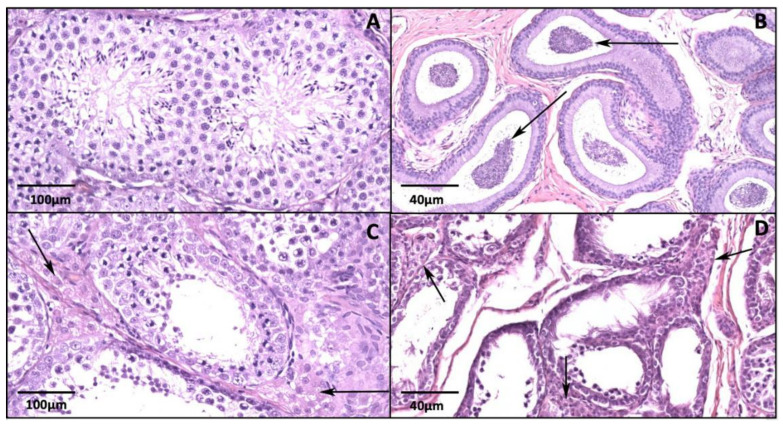
(**A**,**B**) Testicular histology of a cat treated with a 4.7 mg implant. The implant was removed 6 months after insertion and orchiectomy was performed 16 days after the first post-removal serum testosterone rise to >0.1 ng/mL. (**A**) Active testicular parenchyma with normal histology. Seminiferous tubules containing germinal cells (spermatogonia, spermatids, and spermatocytes) variably differentiated toward the lumen. (**B**) Epididymis. Presence of spermatozoa in the tubular lumina (arrows). (**C**) Testicular histology of a cat treated with a 4.7 mg implant. The implant was removed 9 months after insertion and orchiectomy was performed 11 days after the first post-removal serum testosterone rise to >0.1 ng/mL. Testicular parenchyma with a light activity without all lines of spermatogenesis, with no or rare mature elements. Mild increase of small Leydig cells in the interstitium (arrows). (**D**) Testicular histology of a cat treated with a 4.7 mg implant. The implant was removed 9 months after insertion and orchiectomy was performed 7 days after the first post-removal serum testosterone rise to >0.1ng/mL. Multifocal, occasional, and incomplete differentiation of the spermatogonia. The Leydig cells are abundant (arrows) and show mild vacuolization of the cytoplasm. Seminiferous tubules show only immature elements and Sertoli cells, often in a unique row of lining cells. The lumina are empty.

Testicular histology was performed in both testicles’ of 13/15 cats. The owners of the remaining two cats did not request orchiectomy and both cats were treated with another deslorelin implant. Testicular histology showed from limited to normal activity depending on whether orchiectomy was performed during the first week or the second week after resumption of testosterone secretion, respectively (Figure 3A–D). In tomcats in which orchiectomy was performed 10–14 days after the first post-removal rise of serum T, a higher testicular activity was observed, compared to tomcats sterilized during the first 7 days after first post-removal rise of serum T. Moreover, testicular parenchyma of tomcats that were sterilized in the first week after T rise showed an incomplete differentiation of spermatogonia with seminiferous tubules characterized by only immature elements and Sertoli cells without content. In these tomcats, no spermatozoa were observed in the epididymis. When orchiectomy was performed between 10 and 14 days after the rise in T concentration, a variable differentiation of germinal cells (spermatogonia, spermatids, and spermatocytes) in the seminiferous tubules and presence of spermatozoa in the tubular lumen of the epididymis were observed. No differences on testicular activity between left or right testicles was evident.

## 4. Discussion

Orchiectomy is still considered as the elective choice for reproductive control in shelter animals as well as for a relevant percentage of client-owned, companion animals. For animals with breeding value long-acting GnRH agonists such as deslorelin are becoming increasingly considered by pet owners because of their reversible action [3,4,8]. Apart from the induction of reversible sterility, a number of useful clinical applications of deslorelin implants have been also reported in cats such as postponement of puberty, treatment of reproductive diseases and control of behavioral problems due to sex hormones with these effects being dose dependent when comparing the 4.7 mg and 9.4 mg dosage [12,17,18,19,25,26,27,28,29].

This is the first study documenting the interval between early removal of a 4.7 mg deslorelin implant and resumption of full testicular function in tomcats, including a description of the surgical procedure as well as implant characteristics at removal. The main objective of the present study was to assess how long would it take for tomcats treated with a 4.7 mg deslorelin implant for three different time periods (3, 6, and 9 months) to restore their full testicular function. Prior to deslorelin treatment, all subjects presented normal concentration of serum T (6.73 ± 3.41 ng/mL). Once implanted, all tomcats presented behavioral (no roaming, no urine marking), anatomical (disappearance of penile spikes, reduction of testicular volume, and increase in body weight) and endocrinological changes (T < 0.1 ng/mL) when rechecked only once during the treatment and at the end of the treatment period in line with what previously described [12,16,17,19,29,30]. Reduction of testicular volume during deslorelin treatment was 45 ± 27%. Following implant removal, testicular volume increased by a daily rate of 0.17 ± 0.77% from the day of the implant removal until the day when serum T secretion was restored as described in previous studies [12,16].

Implant removal was an easy surgical procedure. In dogs, this practice is usually performed through local application of lidocaine unless the animal is overly excited. As cats may be more difficult to restrain when compared to canine’s, general anesthesia is probably better suited in order to avoid uncoordinated movements during sedation, as these may complicate the procedure causing incomplete removal due to implant rupture.

Following implant removal, the restoration of testosterone secretion occurred between 13 and 28 days and was similar for the three groups of cats with a mean of 23 days and without differences due to photoperiod. In tomcats testosterone secretion is not influenced by season [31], which may explain the lack of difference in the time needed for the hypothalamic-hypophyseal-gonadal axis to regain full function once deslorelin has been cleared from the general circulation. The short interval between implant removal and resumption of testicular function is supported by the first increases in testicular volume being observed 14 days after implant removal. These results suggest that deslorelin was not affecting the hypothalamic-hypophyseal-gonadal axis any longer already 2 weeks after implant removal, perhaps indicating that this is the period necessary for clearance of deslorelin from the general circulation, which further confirms the lack of difference of the interval from implant removal to resumption of testicular function across the three treatment groups.

Even if testosterone secretion was restored approximately 3 weeks following implant removal, no spermatozoa were found on semen collection on the day of orchiectomy. This fact can be associated with the duration of the spermatogenic cycle in the cat which is 46.8 days [31]. Tomcats included in the study were sterilized 2–14 days after a T rise was first detected in serum. Therefore, the finding of no sperm upon semen collection may be due to the fact that spermatogenesis has not yet been completed in the cats of our study by the time of orchiectomy after first serum testosterone detection in blood.

When histology of testicles was evaluated, testicles showed various degrees of spermatogenic activity. Histological data clearly indicate that in deslorelin treated cat’s spermatogenesis resumes rather quickly following implant removal. As observed in the present study, tomcats sterilized in the first week after a rise in testosterone concentration showed a lower degree of testicular activity (incomplete differentiation of spermatozoa and seminiferous tubules featuring only immature elements) when compared to testicles of tomcats sterilized two weeks following the first rise in serum testosterone concentration (in which the complete range of spermatogenic elements were detected in the lumen of seminiferous tubules and mature sperms in the epididymis).

Rupture of deslorelin implants at removal has never been reported. The implant may tend to become friable with time and/or with subcutaneous fat enclosure. Although the degree of implant softening and risk of rupture seemed to increase with increasing length of treatment, no significant association between implant rupture or fat enclosure was observed in the three groups of treated cats (*p* > 0.05). Further studies with larger number of animals may elucidate the mechanism/s laying behind implant rupture. Body weight increased significantly more in cats implanted for 6 and 9 months compared to those treated for 3 months. Although our protocol did not include sham-implanted cats, the increase in body weight with time highlights the potential effect of deslorelin to cause increased body weight when the implant is left in situ for 6 or more months. Further studies on metabolism of deslorelin treated cats would help clarify the mechanism/s behind such an increase in body weight as well as the time needed to return to the initial body weight following the end of treatment. Fat accumulation in the abdominal region occurring during weight increase may contribute together with time to cause softening of the implant thus making its identification as well as rupture during removal procedures more likely.

Not finding a small fragment of a deslorelin implant may frustrate client’s expectations of a tomcat to not return quickly to full testicular activity, as these small pieces may carry a residual action thus prolonging the state of reproductive quiescence. Furthermore, even if implants are found intact in the subcutaneous tissue, manipulation with hemostatic forceps during removal may cause the implant to rupture. Our observations during removal showed that in 69% of the cats of our study a deslorelin implant was found broken in two or more pieces, causing the removal procedure to last longer. The possibility of an incomplete removal procedure may explain the outlier tomcat that did not return to normal T levels as rapidly as the other tomcats.

## 5. Conclusions

Removal of a 4.7 mg deslorelin implant 3, 6, or 9 months after insertion in the subcutaneous tissue of the umbilical region of adult tomcats is followed by restoration of full testicular function after approximately 3 weeks. Deslorelin implants may soften and break with time, although generally this does not prevent a complete removal. The practical implication of this study is that 4.7 mg deslorelin implants may be safely used to shorten the duration of chemical castration in tomcats. Post-treatment fertility was not the object of this study. However, since the regaining of fertility has been demonstrated in tomcats treated with deslorelin for periods of time longer than 9 months [12,17], fertility following an early implant removal is likely to be normal.

## Figures and Tables

**Table 1:** Cat identification, conditions of 4.7 mg deslorelin implants at time of removal, n. of pieces recovered (1 = intact implant) and whether or not implant was enclosed in subcutaneous fat tissue in 15 adult male cats treated with a 4.7 mg deslorelin implant. Implants were removed 3 (green), 6 (orange), or 9 months (grey) following insertion.

Treatment	Cat ID	Ruptured Implant	N. of Recovered Pieces of Implant	Implant Enclosed in Abdominal Fat
	1	-	1	-
	2	-	1	-
	3	X	2	-
**3 months**	4	-	1	X
	5	X	2	-
	6	X	3	X
	7	X	Not available	-
	8	X	5	X
**6 months**	9	-	1	X
	10	-	1	X
	11	X	6	-
	12	X	5	X
**9 months**	13	X	4	X
	14	X	6	X
	15	X	4	-

**Table 2:** Mean testicular volumes (cm^3^) and serum testosterone concentration (ng/mL) in cats treated with a 4.7 mg deslorelin implant at the beginning of the treatment, on the day of implant removal after 3 (green), 6 (orange), and 9 months (grey), on the day of first testosterone rise detection (>0.1 ng/mL), following implant removal and body weights (kg) on the implantation day and day of implant removal.

Treatment	Testicular Volume(cm^3^) at Implantation Day	Testicular Volume(cm^3^) at Implant Removal Day	Testicular Volume(cm^3^) at (T) > 0.1 ng/mL	Decrease (%) of Testicular Volume at Implant Removal	Increase (%) of Testicular Volume at (T) > 0.1 ng/mL Based on Volume after Implant Removal	Body Weight at Implantation (kg)	Body Weight at Removal (kg)	(T) ng/mL at Implantation Day	(T) ng/mL at Implant Removal Day	(T) ng/mL at First Detection Post Implant Removal
	0.87	0.47	1.55	45.97	229.79	4.9	4.82	10.08	<0.1	4.41
	0.91	0.64	1.87	29.67	192.19	5.11	5.21	6.7	<0.1	4.09
	1.39	0.27	0.77	80.57	185.18	4.72	4.82	4.18	<0.1	3.54
**3 months**	2.08	1.5	1.98	27.88	32	4.52	4.63	9.17	<0.1	8.2
	2.43	1.15	1.24	52.67	7.82	4.81	5.12	5.62	< 0.1	4.17
	0.466	0.45	0.66	3.43	46.67	3.4	3.62	4.96	<0.1	0.66
	0.84	0.35	1.1	58.33	214.28	3.10	6.63	5.21	<0.1	3.55
	0.42	0.38	0.5	9.52	31.58	4.52	6.71	3.49	<0.1	2.96
**6 months**	1.66	1.35	1.66	18.67	22.97	4.91	5.5	6.45	<0.1	3.82
	2.48	1.13	1.38	54.43	22.12	4.9	5.82	13.84	<0.1	11.48
	1.26	0.356	0.89	71.74	150	4.22	6.3	4.33	<0.1	5.18
	2.9	0.48	1.39	83.45	189.58	4.21	5.61	4.97	<0.1	2.3
**9 months**	0.65	0.45	1.55	30.77	244.44	3.33	4.22	6.11	<0.1	2.16
	2.35	0.57	1.81	75.74	2.17.54	3.41	4.82	16.2	<0.1	2.21
	1.34	0.49	1.19	63.43	142.86	3.5	6.71	13.4	<0.1	1.99
